# 8-(Naphthalen-1-yl)quinoline

**DOI:** 10.1107/S1600536811034052

**Published:** 2011-08-31

**Authors:** Godwin Kanu, Roger A. Lalancette, Dale E. Vitale

**Affiliations:** a Department of Chemistry and Biochemistry, UCLA, 607 Charles E. Young Drive East, Box 951569, Los Angeles, CA 90095, USA; b Department of Chemistry, Rutgers University-Newark, 73 Warren Street, Newark, NJ 07102-1811, USA; c Department of Chemistry, Kean University, Union, NJ 07083, USA

## Abstract

In the title mol­ecule, C_19_H_13_N, the angle between the mean planes of the naphthalene and quinoline ring systems is 68.59 (2)°. The compound is of inter­est with respect to its potential for spontaneous resolution. In the crystal structure, the *R* and *S* isomers are arranged in alternating homochiral layers. The mol­ecules of a given layer are oriented with their major axes (*i.e.* the axis perpendicular to the interannular bond) in the same direction and their naphthalene and quinoline ring systems are arranged parallel. Like the configurations, this orientation alternates in adjacent layers.

## Related literature

For spontanteous-resolution experiments, see: Asakura & Plasson (2006 ▶); Kondipudi *et al.* (1999 ▶); Kranz *et al.* (1993 ▶); Sainz-Diaz *et al.* (2005 ▶); Wilson & Pincock (1974 ▶). For related structures, see: Kerr & Robertson (1969 ▶); Kuroda & Manson (1981 ▶). For details of the synthesis, see: Huff *et al.* (1998 ▶).
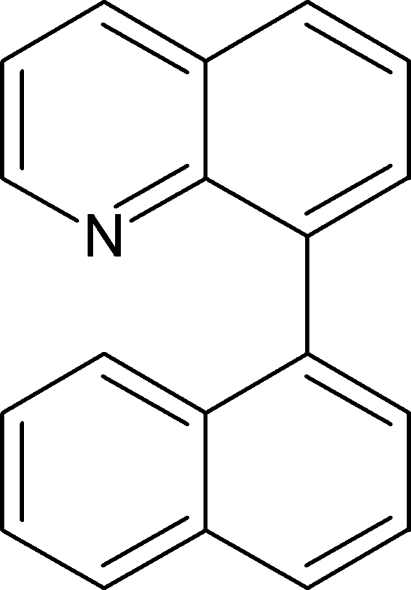


## Experimental

### 

#### Crystal data

C_19_H_13_N
*M* *_r_* = 255.30
Triclinic, 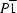

*a* = 6.1778 (1) Å
*b* = 10.0392 (2) Å
*c* = 10.8828 (2) Å
α = 104.537 (1)°
β = 106.435 (1)°
γ = 90.002 (1)°
*V* = 624.80 (2) Å^3^
*Z* = 2
Cu *K*α radiation
μ = 0.61 mm^−1^
*T* = 100 K
0.37 × 0.20 × 0.10 mm

#### Data collection

Bruker SMART CCD APEXII diffractometer
Absorption correction: numerical (*SADABS*; Sheldrick, 2008 ▶) *T*_min_ = 0.806, *T*_max_ = 0.942
5783 measured reflections
2058 independent reflections
1678 reflections with *I* > 2σ(*I*)
*R*_int_ = 0.011

#### Refinement

*R*[*F* ^2^ > 2σ(*F* ^2^)] = 0.044
*wR*(*F* ^2^) = 0.131
*S* = 1.06
2058 reflections
181 parameters
H-atom parameters constrained
Δρ_max_ = 0.38 e Å^−3^
Δρ_min_ = −0.34 e Å^−3^

### 

Data collection: *APEX2* (Bruker, 2006 ▶); cell refinement: *APEX2*; data reduction: *SAINT* (Bruker, 2005 ▶); program(s) used to solve structure: *SHELXTL* (Sheldrick, 2008 ▶); program(s) used to refine structure: *SHELXTL*; molecular graphics: *Mercury* (Macrae *et al.*, 2008 ▶); software used to prepare material for publication: *SHELXTL*.

## Supplementary Material

Crystal structure: contains datablock(s) I, global. DOI: 10.1107/S1600536811034052/lh5298sup1.cif

Supplementary material file. DOI: 10.1107/S1600536811034052/lh5298Isup2.mol

Structure factors: contains datablock(s) I. DOI: 10.1107/S1600536811034052/lh5298Isup3.hkl

Supplementary material file. DOI: 10.1107/S1600536811034052/lh5298Isup4.cml

Additional supplementary materials:  crystallographic information; 3D view; checkCIF report

## Figures and Tables

**Fig. 1.**
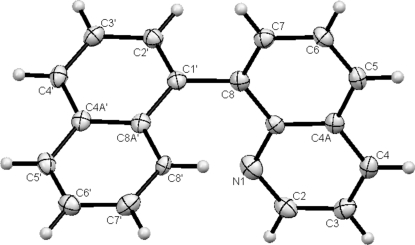
The molecular structure of the S enantiomer of (I) with atom numbering scheme. Displacement ellipsoids are drawn at 50% probability level.

**Fig. 2.**
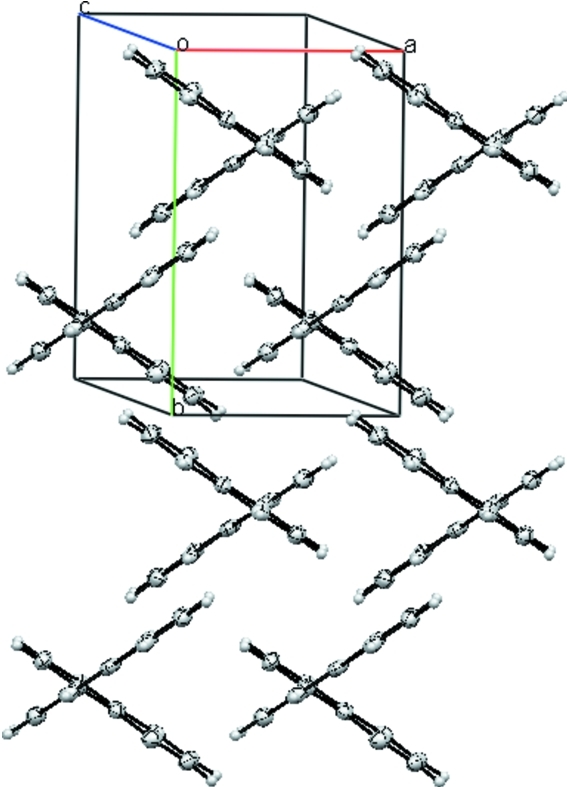
Alternating layers of R and S isomers viewed along c axis; R, S, R, S from top of figure.

## supplementary crystallographic information

### Comment

The structural similarities between the title compound (81QNNP) and 1,1'-binaphthalenyl (11BNP) suggest that, like the hydrocarbon, 81QNNP might exhibit spontaneous resolution. The characteristics that afford spontaneous resolution of the racemic compound of 11BNP are its dimorphism and moderate barrier to rotation about the C1—C1' interannular bond (Kranz *et al.*, 1993). The barrier is large enough to prevent racemization of resolved forms below about 351 K, but small enough to afford rapid interconversion of the enantiomers in the melt. The dimorphs consist of an optically inactive racemic compound (Kerr & Robertson, 1969) that is the more stable form at temperatures below 351 K and a conglomerate of single crystals of the *R* and S isomers (Kuroda & Manson, 1981) that is the more stable above this temperature. Accordingly, heating of the racemic compound above the melting point of the conglomerate (431 K) followed by supercooling to 423 K can produce an optically active solid (Kondipudi *et al.*, 1999).

The molecular structure of the title compound is shown in Fig. 1. The title compound closely resembles 1,1-binaphthalenyl in molecular structure, crystal structure, and thermal behavior (Wilson & Pincock, 1974). On the molecular level, the two compounds differ only in the substitution of a nitrogen in 81QNNP for the C8 carbon in 11BNP. Likewise, the room temperature solid of 81QNNP is an optically inactive racemic compound with the components of an enantiotopic pair of molecules in the unit cell. Moreover, while spontaneous development of optical activity has not been demonstrated in the title compound, preliminary DSC results suggest that it is polymorphic. This means that, in addition to the racemic compound described herein, 81QNNP may also exist as a conglomerate. If so, it has the potential for spontaneous resolution *via* a mechanism similar to that of 11BNP (Asakura & Plasson, 2006; Sainz-Diaz *et al.*, 2005). In the crystal, the *R* and *S* isomers are arranged in alternating homochiral layers. The molecules of a given layer are oriented with their major axes in the same direction and their naphthalene and quinoline ring systems are arranged parallel. Like the configurations this orientation alternates in adjacent layers (Fig. 2).

### Experimental

The synthesis was carried out according to a literature procedure (Huff *et al.*, 1998). 8-(Naphthalen-1-yl)quinoline was synthesized in 58% yield from 8-bromoquinoline (Frontier Chemical) and 1-naphthalenylboronic acid (Sigma–Aldrich) using a modification of the Suzuki coupling reaction (Huff *et al.*, 1998) and crystallized from 1-propanol; m.p. 436.0–437.5 K, ^13^C NMR (CDCl_3_): DEPT-CH δ 121.3, 125.6, 125.9, 126.0, 126.4, 127.0, 128.1, 128.2, 128.3, 128.6, 131.9, 136.4, 150.9 p.p.m. IR (KBr ν cm^-1^): 3041 (CH_ar_) 1592 and 1491 (C═C and C═N), 1379, 1310, 1204, 1064, 1015, 944, 828, 797, 782, 772, 677, 617, 517.

### Refinement

All H atoms for were found in electron density difference maps. These were placed in geometrically idealized positions and constrained to ride on their parent C atoms with C—H distances of 0.95 Å, and *U*_iso_(H) = 1.2*U*_eq_(C).

### Crystal data

**Table:**

C_19_H_13_N	*Z* = 2
*M**_r_* = 255.30	*F*(000) = 268
Triclinic, *P*1	*D*_x_ = 1.357 Mg m^−^^3^
Hall symbol: -P 1	Melting point = 436.0–437.5 K
*a* = 6.1778 (1) Å	Cu *K*α radiation, λ = 1.54178 Å
*b* = 10.0392 (2) Å	Cell parameters from 3661 reflections
*c* = 10.8828 (2) Å	θ = 4.4–66.7°
α = 104.537 (1)°	µ = 0.61 mm^−^^1^
β = 106.435 (1)°	*T* = 100 K
γ = 90.002 (1)°	Plate, colourless
*V* = 624.80 (2) Å^3^	0.37 × 0.20 × 0.10 mm

### Data collection

**Table:**

Bruker SMART CCD APEXII diffractometer	2058 independent reflections
Radiation source: fine-focus sealed tube	1678 reflections with *I* > 2σ(*I*)
graphite	*R*_int_ = 0.011
φ and ω scans	θ_max_ = 67.5°, θ_min_ = 4.4°
Absorption correction: numerical (*SADABS*; Sheldrick, 2008)	*h* = −7→7
*T*_min_ = 0.806, *T*_max_ = 0.942	*k* = −11→11
5783 measured reflections	*l* = −12→12

### Refinement

**Table:**

Refinement on *F*^2^	Primary atom site location: structure-invariant direct methods
Least-squares matrix: full	Secondary atom site location: difference Fourier map
*R*[*F*^2^ > 2σ(*F*^2^)] = 0.044	Hydrogen site location: inferred from neighbouring sites
*wR*(*F*^2^) = 0.131	H-atom parameters constrained
*S* = 1.06	*w* = 1/[σ^2^(*F*_o_^2^) + (0.0654*P*)^2^ + 0.348*P*] where *P* = (*F*_o_^2^ + 2*F*_c_^2^)/3
2058 reflections	(Δ/σ)_max_ < 0.001
181 parameters	Δρ_max_ = 0.38 e Å^−^^3^
0 restraints	Δρ_min_ = −0.34 e Å^−^^3^

### Special details

**Table:**

Experimental. crystal mounted on a Cryoloop using Paratone-N
Geometry. All e.s.d.'s (except the e.s.d. in the dihedral angle between two l.s. planes) are estimated using the full covariance matrix. The cell e.s.d.'s are taken into account individually in the estimation of e.s.d.'s in distances, angles and torsion angles; correlations between e.s.d.'s in cell parameters are only used when they are defined by crystal symmetry. An approximate (isotropic) treatment of cell e.s.d.'s is used for estimating e.s.d.'s involving l.s. planes.
Refinement. Refinement of *F*^2^ against ALL reflections. The weighted *R*-factor *wR* and goodness of fit *S* are based on *F*^2^, conventional *R*-factors *R* are based on *F*, with *F* set to zero for negative *F*^2^. The threshold expression of *F*^2^ > σ(*F*^2^) is used only for calculating *R*-factors(gt) *etc*. and is not relevant to the choice of reflections for refinement. *R*-factors based on *F*^2^ are statistically about twice as large as those based on *F*, and *R*-factors based on ALL data will be even larger.

### Fractional atomic coordinates and isotropic or equivalent isotropic displacement parameters (Å^2^)

**Table:**

	*x*	*y*	*z*	*U*_iso_*/*U*_eq_	
N1	0.9761 (3)	0.89896 (16)	1.10848 (15)	0.0304 (4)	
C2	1.1692 (3)	0.96964 (18)	1.19213 (17)	0.0269 (4)	
H2	1.2610	1.0176	1.1579	0.032*	
C3	1.2400 (3)	0.97528 (18)	1.32817 (17)	0.0268 (4)	
H3	1.3785	1.0249	1.3839	0.032*	
C4	1.1084 (3)	0.90897 (17)	1.37947 (16)	0.0233 (4)	
H4	1.1549	0.9126	1.4713	0.028*	
C4A	0.9019 (3)	0.83446 (16)	1.29589 (15)	0.0207 (4)	
C5	0.7566 (3)	0.76657 (17)	1.34510 (16)	0.0230 (4)	
H5	0.7968	0.7702	1.4369	0.028*	
C6	0.5598 (3)	0.69612 (17)	1.26156 (16)	0.0243 (4)	
H6	0.4636	0.6510	1.2954	0.029*	
C7	0.4982 (3)	0.68992 (17)	1.12520 (16)	0.0236 (4)	
H7	0.3602	0.6406	1.0684	0.028*	
C8	0.6333 (3)	0.75369 (17)	1.07236 (16)	0.0211 (4)	
C8A	0.8399 (3)	0.82928 (16)	1.15855 (15)	0.0197 (4)	
C1'	0.5605 (3)	0.74639 (17)	0.92755 (16)	0.0210 (4)	
C2'	0.3732 (3)	0.81036 (17)	0.87514 (16)	0.0234 (4)	
H2'	0.2924	0.8601	0.9321	0.028*	
C3'	0.2981 (3)	0.80380 (17)	0.73857 (16)	0.0244 (4)	
H3'	0.1677	0.8487	0.7048	0.029*	
C4'	0.4114 (3)	0.73338 (17)	0.65465 (16)	0.0229 (4)	
H4'	0.3598	0.7297	0.5628	0.027*	
C4A'	0.6059 (3)	0.66569 (16)	0.70394 (15)	0.0207 (4)	
C5'	0.7289 (3)	0.59114 (17)	0.62054 (16)	0.0234 (4)	
H5'	0.6837	0.5877	0.5287	0.028*	
C6'	0.9118 (3)	0.52436 (18)	0.67111 (17)	0.0263 (4)	
H6'	0.9937	0.4743	0.6150	0.032*	
C7'	0.9774 (3)	0.53047 (18)	0.80697 (17)	0.0274 (4)	
H7'	1.1039	0.4830	0.8413	0.033*	
C8'	0.8677 (2)	0.60111 (16)	0.89035 (15)	0.0173 (4)	
H8'	0.9166	0.6033	0.9819	0.021*	
C8A'	0.6816 (3)	0.67103 (16)	0.84112 (15)	0.0204 (4)	

### Atomic displacement parameters (Å^2^)

**Table:**

	*U*^11^	*U*^22^	*U*^33^	*U*^12^	*U*^13^	*U*^23^
N1	0.0366 (9)	0.0240 (8)	0.0316 (8)	0.0013 (6)	0.0141 (7)	0.0046 (6)
C2	0.0307 (9)	0.0211 (9)	0.0313 (9)	−0.0011 (7)	0.0153 (8)	0.0041 (7)
C3	0.0238 (9)	0.0213 (9)	0.0309 (10)	0.0001 (7)	0.0059 (7)	0.0016 (7)
C4	0.0276 (9)	0.0201 (9)	0.0197 (8)	0.0061 (7)	0.0044 (7)	0.0039 (7)
C4A	0.0249 (8)	0.0154 (9)	0.0210 (8)	0.0051 (6)	0.0070 (7)	0.0034 (6)
C5	0.0314 (9)	0.0201 (9)	0.0191 (8)	0.0057 (7)	0.0093 (7)	0.0058 (6)
C6	0.0285 (9)	0.0217 (9)	0.0261 (9)	0.0020 (7)	0.0128 (7)	0.0071 (7)
C7	0.0226 (8)	0.0221 (9)	0.0250 (9)	0.0006 (6)	0.0064 (7)	0.0049 (7)
C8	0.0240 (8)	0.0173 (9)	0.0220 (9)	0.0042 (6)	0.0074 (7)	0.0043 (6)
C8A	0.0227 (8)	0.0163 (9)	0.0211 (8)	0.0039 (6)	0.0085 (7)	0.0043 (6)
C1'	0.0215 (8)	0.0195 (9)	0.0214 (8)	−0.0026 (6)	0.0061 (7)	0.0046 (6)
C2'	0.0236 (8)	0.0229 (9)	0.0235 (9)	0.0015 (7)	0.0082 (7)	0.0043 (7)
C3'	0.0224 (8)	0.0230 (10)	0.0258 (9)	0.0017 (7)	0.0024 (7)	0.0081 (7)
C4'	0.0272 (9)	0.0209 (9)	0.0189 (8)	−0.0029 (7)	0.0035 (7)	0.0060 (6)
C4A'	0.0237 (8)	0.0162 (9)	0.0211 (8)	−0.0036 (6)	0.0046 (7)	0.0052 (6)
C5'	0.0295 (9)	0.0213 (9)	0.0197 (8)	−0.0041 (7)	0.0079 (7)	0.0051 (7)
C6'	0.0274 (9)	0.0225 (10)	0.0288 (9)	0.0004 (7)	0.0116 (7)	0.0028 (7)
C7'	0.0247 (9)	0.0210 (10)	0.0323 (10)	0.0024 (7)	0.0025 (7)	0.0061 (7)
C8'	0.0193 (8)	0.0148 (8)	0.0147 (7)	0.0006 (6)	0.0011 (6)	0.0026 (6)
C8A'	0.0222 (8)	0.0159 (9)	0.0217 (8)	−0.0033 (6)	0.0048 (7)	0.0044 (6)

### Geometric parameters (Å, °)

**Table:**

N1—C2	1.348 (2)	C1'—C2'	1.374 (2)
N1—C8A	1.395 (2)	C1'—C8A'	1.434 (2)
C2—C3	1.406 (2)	C2'—C3'	1.410 (2)
C2—H2	0.9500	C2'—H2'	0.9500
C3—C4	1.364 (2)	C3'—C4'	1.364 (2)
C3—H3	0.9500	C3'—H3'	0.9500
C4—C4A	1.420 (2)	C4'—C4A'	1.418 (2)
C4—H4	0.9500	C4'—H4'	0.9500
C4A—C5	1.418 (2)	C4A'—C5'	1.418 (2)
C4A—C8A	1.421 (2)	C4A'—C8A'	1.419 (2)
C5—C6	1.363 (2)	C5'—C6'	1.364 (2)
C5—H5	0.9500	C5'—H5'	0.9500
C6—C7	1.409 (2)	C6'—C7'	1.403 (2)
C6—H6	0.9500	C6'—H6'	0.9500
C7—C8	1.376 (2)	C7'—C8'	1.346 (2)
C7—H7	0.9500	C7'—H7'	0.9500
C8—C8A	1.432 (2)	C8'—C8A'	1.393 (2)
C8—C1'	1.494 (2)	C8'—H8'	0.9500
			
C2—N1—C8A	118.89 (15)	C2'—C1'—C8	120.02 (15)
N1—C2—C3	122.60 (15)	C8A'—C1'—C8	120.85 (14)
N1—C2—H2	118.7	C1'—C2'—C3'	121.44 (15)
C3—C2—H2	118.7	C1'—C2'—H2'	119.3
C4—C3—C2	119.48 (15)	C3'—C2'—H2'	119.3
C4—C3—H3	120.3	C4'—C3'—C2'	120.43 (15)
C2—C3—H3	120.3	C4'—C3'—H3'	119.8
C3—C4—C4A	120.15 (15)	C2'—C3'—H3'	119.8
C3—C4—H4	119.9	C3'—C4'—C4A'	120.27 (15)
C4A—C4—H4	119.9	C3'—C4'—H4'	119.9
C5—C4A—C4	122.25 (15)	C4A'—C4'—H4'	119.9
C5—C4A—C8A	119.47 (15)	C5'—C4A'—C4'	122.28 (15)
C4—C4A—C8A	118.27 (15)	C5'—C4A'—C8A'	118.09 (15)
C6—C5—C4A	120.44 (15)	C4'—C4A'—C8A'	119.63 (15)
C6—C5—H5	119.8	C6'—C5'—C4A'	120.54 (15)
C4A—C5—H5	119.8	C6'—C5'—H5'	119.7
C5—C6—C7	120.34 (15)	C4A'—C5'—H5'	119.7
C5—C6—H6	119.8	C5'—C6'—C7'	119.23 (15)
C7—C6—H6	119.8	C5'—C6'—H6'	120.4
C8—C7—C6	121.54 (15)	C7'—C6'—H6'	120.4
C8—C7—H7	119.2	C8'—C7'—C6'	122.44 (15)
C6—C7—H7	119.2	C8'—C7'—H7'	118.8
C7—C8—C8A	119.05 (15)	C6'—C7'—H7'	118.8
C7—C8—C1'	119.94 (15)	C7'—C8'—C8A'	119.33 (15)
C8A—C8—C1'	120.99 (14)	C7'—C8'—H8'	120.3
N1—C8A—C4A	120.58 (15)	C8A'—C8'—H8'	120.3
N1—C8A—C8	120.26 (14)	C8'—C8A'—C4A'	120.35 (15)
C4A—C8A—C8	119.15 (15)	C8'—C8A'—C1'	120.53 (14)
C2'—C1'—C8A'	119.12 (15)	C4A'—C8A'—C1'	119.10 (15)
			
C8A—N1—C2—C3	0.4 (3)	C7—C8—C1'—C8A'	−112.47 (18)
N1—C2—C3—C4	−1.1 (3)	C8A—C8—C1'—C8A'	69.1 (2)
C2—C3—C4—C4A	0.2 (2)	C8A'—C1'—C2'—C3'	−0.2 (2)
C3—C4—C4A—C5	−178.48 (15)	C8—C1'—C2'—C3'	−179.08 (15)
C3—C4—C4A—C8A	1.1 (2)	C1'—C2'—C3'—C4'	−0.2 (2)
C4—C4A—C5—C6	179.99 (15)	C2'—C3'—C4'—C4A'	0.1 (2)
C8A—C4A—C5—C6	0.4 (2)	C3'—C4'—C4A'—C5'	179.92 (15)
C4A—C5—C6—C7	0.0 (2)	C3'—C4'—C4A'—C8A'	0.3 (2)
C5—C6—C7—C8	0.2 (3)	C4'—C4A'—C5'—C6'	−178.28 (15)
C6—C7—C8—C8A	−0.7 (2)	C8A'—C4A'—C5'—C6'	1.4 (2)
C6—C7—C8—C1'	−179.14 (15)	C4A'—C5'—C6'—C7'	−0.1 (2)
C2—N1—C8A—C4A	1.0 (2)	C5'—C6'—C7'—C8'	−0.6 (3)
C2—N1—C8A—C8	179.75 (15)	C6'—C7'—C8'—C8A'	0.1 (2)
C5—C4A—C8A—N1	177.87 (14)	C7'—C8'—C8A'—C4A'	1.2 (2)
C4—C4A—C8A—N1	−1.7 (2)	C7'—C8'—C8A'—C1'	179.58 (14)
C5—C4A—C8A—C8	−0.9 (2)	C5'—C4A'—C8A'—C8'	−1.9 (2)
C4—C4A—C8A—C8	179.47 (14)	C4'—C4A'—C8A'—C8'	177.77 (14)
C7—C8—C8A—N1	−177.73 (14)	C5'—C4A'—C8A'—C1'	179.69 (13)
C1'—C8—C8A—N1	0.7 (2)	C4'—C4A'—C8A'—C1'	−0.7 (2)
C7—C8—C8A—C4A	1.1 (2)	C2'—C1'—C8A'—C8'	−177.81 (14)
C1'—C8—C8A—C4A	179.50 (14)	C8—C1'—C8A'—C8'	1.1 (2)
C7—C8—C1'—C2'	66.4 (2)	C2'—C1'—C8A'—C4A'	0.6 (2)
C8A—C8—C1'—C2'	−112.04 (18)	C8—C1'—C8A'—C4A'	179.48 (14)
